# Interfacial Proton‐Relay Microenvironment Enables Self‐Driven Singlet Oxygen Generation under Neutral Conditions

**DOI:** 10.1002/advs.76027

**Published:** 2026-06-10

**Authors:** Qiaoyu Gao, Xiaohui Dai, Jian Ye, Lili Li, Chenxiao Yu, Yuehan Jiang, Jiangdong Dai, Xiaohua Tian, Jun Zhao, Jianming Pan

**Affiliations:** ^1^ School of Chemistry and Chemical Engineering Jiangsu University Zhenjiang China; ^2^ Applied Research Centre for Pearl River Delta Environment Institute of Advanced Materials, Department of Biology Hong Kong Baptist University Kowloon Tong Hong Kong SAR China

**Keywords:** acidic microenvironment, molecular oxygen, neutral condition, proton relay, singlet oxygen

## Abstract

The energy‐free activation of ambient molecular oxygen (O_2_) to singlet oxygen (^1^O_2_) under neutral conditions is highly desirable for green oxidation chemistry, yet remains fundamentally limited by sluggish proton‐coupled *OOH formation and desorption. Here, we engineer an interfacial proton‐relay microenvironment between MoS_2_ and CuCl that enables self‐driven O_2_‐to‐^1^O_2_ conversion without external energy inputs. Electron‐deficient sulfur sites act as a proton reservoir by forming S‐H_ads_ species, facilitating directional proton migration through Cu‐S‐Mo channels to activate adsorbed O_2_ on electron‐rich Cu sites. This coupled electron‐proton relay accelerates *OOH hydrogenation while maintaining moderate *O2/*OOH binding, effectively suppressing O─O bond cleavage and favoring a 1O2‐dominated pathway. As a result, the system achieves quantitative pollutant removal and sustained operation for over 16 h in pilot‐scale membrane filtration. This interfacial design is broadly applicable to transition metal sulfides, offering a general strategy to overcome proton‐transfer limitations and advance autonomous catalytic platforms for sustainable oxidation and environmental remediation.

## Graphical abstract

An interfacial proton‐relay microenvironment at MoS_2_‐CuCl heterostructures enables selective O_2_ activation to ^1^O_2_ under neutral conditions. Electron‐deficient sulfur sites mediate proton transfer to Cu‐bound O_2_, accelerating *OOH hydrogenation while suppressing O─O bond cleavage, achieving stable, efficient pollutant removal.

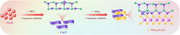

## 1 Introduction

Advanced oxidation processes (AOPs) harnessing reactive oxygen species (ROS) underpin modern water decontamination strategies, yet remain fundamentally constrained by an intractable compromise between oxidation capacity and selectivity [1]. Despite the formidable redox potentials of hydroxyl radicals (•OH, 1.9–2.7 V vs NHE), their nonselective reactivity and ultrashort lifetimes (<40 µs) lead to poor utilization efficiency and irreversible catalyst fouling, limitations that persist even in state‐of‐the‐art AOPs [2]. In contrast, singlet oxygen (^1^O_2_) offers a compelling alternative due to its mild oxidation ability, prolonged lifetime (86 µs), and selectivity toward electron‐rich aromatic pollutants via electrophilic addition [3, 4]. However, efficient ^1^O_2_ synthesis remains challenging, often relying on excessive chemical oxidants (e.g., hydrogen peroxide (H_2_O_2_), peroxymonosulfate (PMS), and peroxydisulfate (PDS)) or energy inputs (light, electricity) with poor selectivity control [5, 6]. Given Earth's atmosphere contains ∼21% O_2_, this abundant resource offers the most green and cost‐effective pathway for ambient ^1^O_2_ synthesis [7]. Yet, spontaneous O_2_‐to‐^1^O_2_ conversion faces a fundamental kinetic barrier under neutral conditions, namely a proton‐deficient environment that impedes the critical protonation step (*O_2_ + H^+^ + e^−^ → *OOH) [8]. This step is essential for generating free O_2_^•−^/•OOH intermediates, which subsequently recombine to yield ^1^O_2_ via disproportionation [9]. Therefore, the key challenge lies in engineering catalysts that can simultaneously supply protons and activate O_2_ under neutral pH, without external energy.

Interfacial acid‐like microenvironment engineering presents a promising strategy for achieving self‐sustaining proton storage and supply capabilities [10, 11, 12]. Studies have demonstrated that sulfur sites in transition metal sulfides (TMS) can efficiently capture H_ads_ from interfacial water molecules by forming S‐H_ads_ bonds, acting as a proton “sponge” and establishing localized proton‐enriched microdomains [13, 14]. These confined protons can dynamically shuttle to adjacent catalytic sites, generating transient H_3_O^+^ species that facilitate rapid proton transfer to adsorbed O_2_, thereby driving *OOH formation via a dynamic proton capture‐relay mechanism, even under neutral bulk conditions [15]. TMS often suffers from poor O_2_ activation kinetics. Conversely, copper(I)‐based catalysts, with a notable O_2_ activation rate constant of 3.1 × 10^4^ M^−1^·s^−1^, exhibit distinctive potential due to their d‐electron‐mediated tunability for O_2_ adsorption, offering an ideal platform for kinetically favorable *O_2_ hydrogenation [13, 16, 17]. Yet, monometallic Cu(I) species are notoriously susceptible to rapid oxidative deactivation in oxygen‐rich Fenton‐like environments, severely limiting their practical application [18, 19, 20]. This inherent instability creates a critical gap in that​ TMS offer superior proton management but often lack the optimal O_2_ activation kinetics of Cu(I), while Cu(I) alone suffers from deactivation and lacks a sufficient proton reservoir under neutral conditions. Consequently, we speculated that constructing covalent Cu‐S‐Mo bridges between MoS_2_ and Cu(I) species interfaces will synergistically drive the simultaneous optimization of free •OOH generation kinetics and robust proton regulation, overcoming the limitations of each component to enable efficient, self‐sustained neutral ^1^O_2_ synthesis.

**FIGURE 1:**
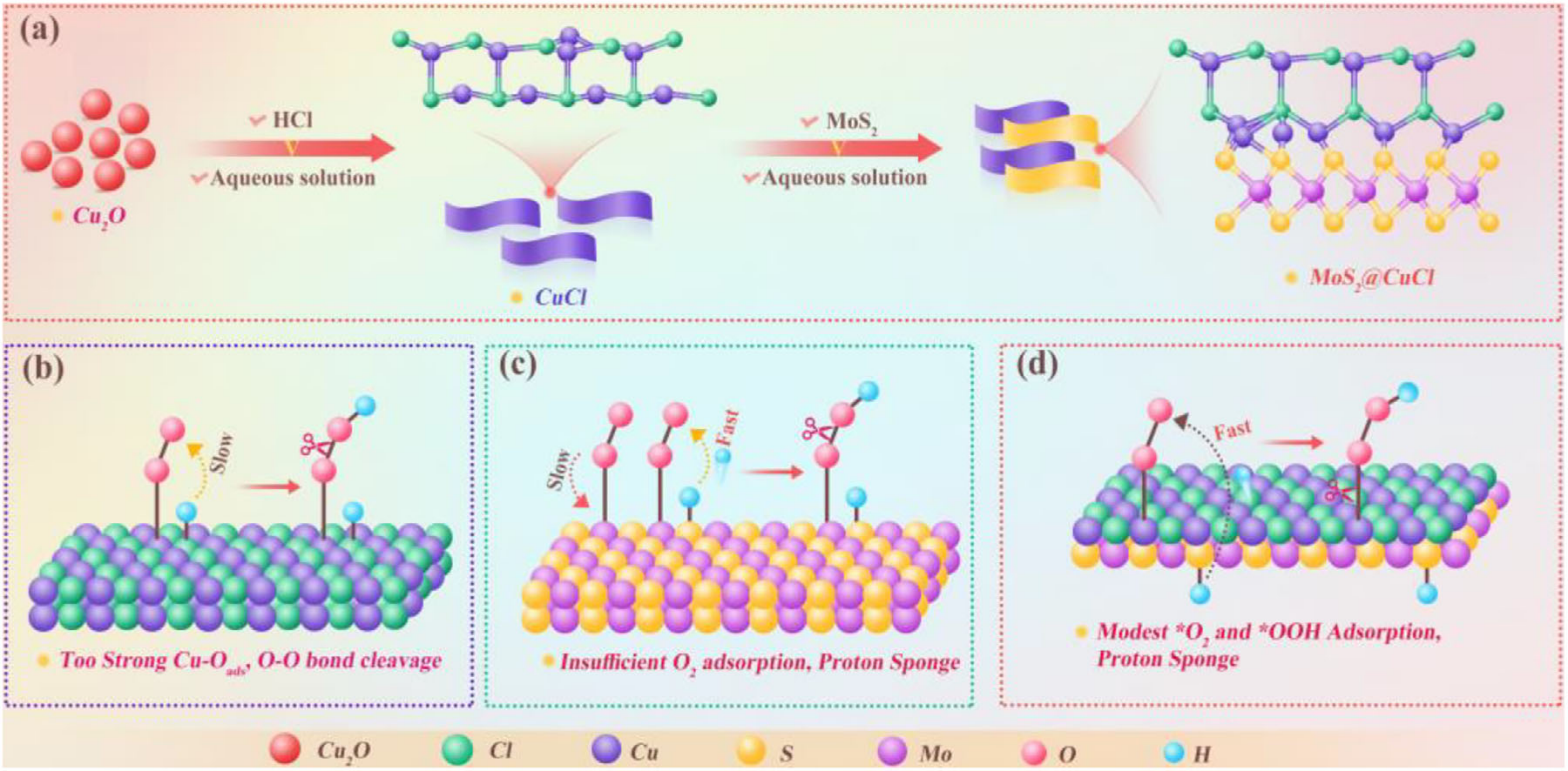
(a) Fabrication process of MoS_2_@CuCl, and (b–d) the summarized performance difference mechanism of O_2_ activation over CuCl, MoS_2_, and MoS_2_@CuCl.

Herein, we propose a strategy that moves away from external energy‐dependent activation toward microenvironment‐driven spontaneous conversion. By constructing a covalent Cu‐S‐Mo bridge between MoS_2_ and CuCl, we create an interfacial acidic microdomain that functions as an integrated proton pump and electron regulator, achieving autonomous O_2_‐to‐^1^O_2_ conversion under neutral conditions without any energy input (Figure 1a). The Cu‐S‐Mo interface provides a continuous proton relay pathway from MoS_2_ to Cu active sites, while stabilizing Cu(I) against oxidation. Experimental and theoretical analyses demonstrate that this tailored microenvironment accelerates the hydrogenation kinetics of *O_2_ to *OOH, moderates *OOH binding strength to prevent O─O bond cleavage, and promotes *OOH desorption, thereby steering the pathway toward ^1^O_2_ generation (Figure 1b). This microenvironment‐driven electron‐proton relay system achieves ultrafast ^1^O_2_‐dominated oxidation of refractory pollutants and maintains exceptional catalytic stability for over 16 h in continuous pilot‐scale membrane filtration. This work resolves the long‐standing proton‐availability bottleneck in neutral ^1^O_2_ synthesis and establishes a design principle for sustainable oxidant generation and water purification.

## 2 Result and Discussion

**FIGURE 2:**
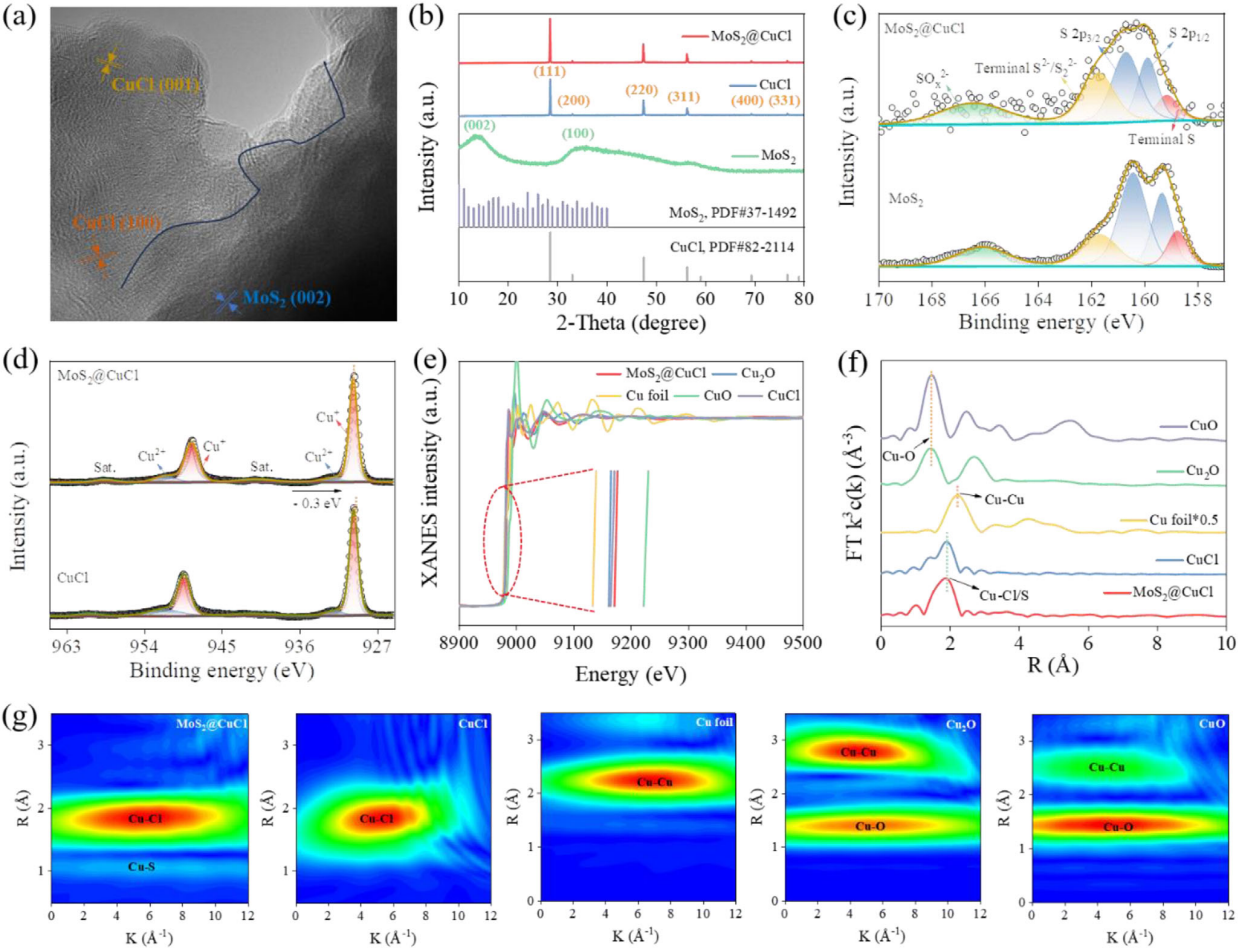
(a) HRTEM image of MoS_2_@CuCl, (b) XRD pattern, XPS spectra of (c) S 2p and (d) Cu 2p, (e) XANES spectra at the Cu K‐edge, (f) FT k^3^‐weighted EXAFS spectra of MoS_2_@CuCl and references, and (g) WT‐EXAFS plots of MoS_2_@CuCl and references.

To elucidate the microstructure of MoS_2_@CuCl, transmission electron microscopy (TEM) analysis was conducted. The TEM images show CuCl grown on the surface of MoS_2_ nanosheets, forming an atomically sharp and intimate interface (Figure 2a; Figure S1). The MoS_2_ support maintained its characteristic layered structure with an interlayer spacing of 0.617 nm (JCPDS No. 37–1492), providing abundant edge‐unsaturated sulfur sites [21], while the observed lattice fringes of 0.393 and 0.395 nm corresponded to the (100) and (001) planes of CuCl (JCPDS No. 82–2114), respectively (Figure S2). Selected Area Electron Diffraction (SAED) was performed to further confirm the crystal phases (Figure S1). Notably, the seamless integration at the boundary region suggested the formation of covalent interactions between the two components. The elemental mapping analysis (Figure S3) confirmed the uniform distribution of Cu, Cl, Mo, and S, indicating the homogeneous growth of CuCl particles on the MoS_2_ nanosheets. As shown in Figure 2b and Figure S4, the characteristic peaks of CuCl could be observed, but MoS_2_ could not be observed in the X‐ray diffraction patterns (XRD) of MoS_2_@CuCl. This is likely due to the high dispersion of MoS_2_ nanosheets and the dominant X‐ray scattering from the CuCl coating, and the possible amorphous or poorly crystalline nature of MoS_2_ under our synthesis conditions, all consistent with the formation of an intimate interfacial coupling between the two components. In combination with Raman spectra (Figure S4), the above characterizations demonstrated that the MoS_2_@CuCl had been obtained and there was a seamless heterojunction interface between CuCl and MoS_2_, which could favor charge transfer.

To gain deeper insights into the electronic structure and interfacial interactions in the MoS_2_@CuCl composite, X‐ray photoelectron spectroscopy (XPS) and synchrotron radiation‐based X‐ray absorption spectroscopy (XAS) analyses were systematically conducted. The XPS survey spectrum confirmed the coexistence of Cu, Cl, S, and Mo elements, suggesting the successful integration of both components (Figure S5). Regarding the S 2p XPS spectrum of MoS_2_ in Figure 2c, the peaks at 159.3 and 160.4 eV reflected S 2p_1/2_ and S 2p_3/2_, respectively. Compared with MoS_2_, the characteristic XPS peak strengths of terminal S_2_^2−^/S^2−^ (edge S, 161.7 eV) and the polysulfides (terminal S, 159.1 eV) of MoS_2_@CuCl significantly increased. This result indicated that the chemical combination of CuCl mainly occurred at the edge of the MoS_2_ nanosheets, significantly changing the edge S of MoS_2_, which was also completely consistent with the TEM results (Figure 2a; Figure S1). Furthermore, distinct binding energy shifts were detected in the Cu 2p, Mo 3d, and Cl 2p spectra of MoS_2_@CuCl relative to the individual counterparts (Figure 2d; Figure S5), providing strong evidence for substantial electron redistribution and a robust interfacial interaction between MoS_2_ and CuCl. This in situ assembly promoted the formation of specific Cu─S covalent bonds, which were crucial for enhancing both electron transfer efficiency and structural stability. Consistently, electrochemical impedance spectroscopy demonstrated a significantly lower charge transfer resistance for MoS_2_@CuCl compared to pure CuCl (Figure S6), underscoring the beneficial role of the asymmetric Cu‐S‐Mo sites in facilitating interfacial electron transfer.

The chemical state and coordination environment of Cu were further elucidated by X‐ray absorption fine structure (XAFS) measurements. The Cu K‐edge X‐ray absorption near‐edge structure (XANES) spectrum of MoS_2_@CuCl, compared with standard references such as Cu foil, Cu_2_O, CuO, and CuCl, is presented in Figure 2e. The Cu K‐edge XANES of MoS_2_@CuCl closely resembled that of Cu_2_O (Figure 2e, inset), indicating that the average valence state of Cu was predominantly +1 [22]. Notably, the absorption edge of MoS_2_@CuCl appeared at a slightly lower energy than that of pure CuCl, implying the presence of a covalent bond between Cu and non‑metal atoms (likely S) in the composite [23, 24]. Additionally, the Fourier‐transformed k_3_‐weighted Cu K‐edge XAFS spectra were employed to further elucidate the coordination environment of Cu in MoS_2_@CuCl (Figure 2f). In contrast to the Cu─Cu bond observed at approximately 2.30 Å in Cu foil, the related XAFS spectra in R space for MoS_2_@CuCl exhibited a primary peak at around 1.87 Å, which had a slight shift to lower energy compared to CuCl, indicating the co‐presence of Cu─S coordination (Figure S7) [25]. Furthermore, the intensity of the Cu‐Cl peak at ∼1.90 Å was higher in MoS_2_@CuCl than in pure CuCl, corroborating the successful integration of MoS_2_ into the heterostructure and suggesting an increased coordination number around Cu. The Cu K‐edge wavelet transform (WT)‐EXAFS analysis also highlighted minor differences in MoS_2_@CuCl and CuCl, confirming the existence of Cu‐S‐Mo covalent bond in MoS_2_@CuCl (Figure 2g) [25]. EXAFS fitting analysis was employed to quantitatively extract the structural parameters of the Cu species, and the fitted curves matched quite well with the experimental spectra. The fitting results give a coordination number (CN) of 3.3 for Cu atoms, with each Cu directly bonded to three S atoms (Figure 2h; Figures S7 and S8 and Table S1). Overall, these results unambiguously confirmed the successful self‐assembly of MoS_2_ and CuCl into an asymmetric Cu‐S‐Mo interfacial structure, which served as the fundamental active site for the enhanced catalytic performance observed in subsequent experiments.

**FIGURE 3:**
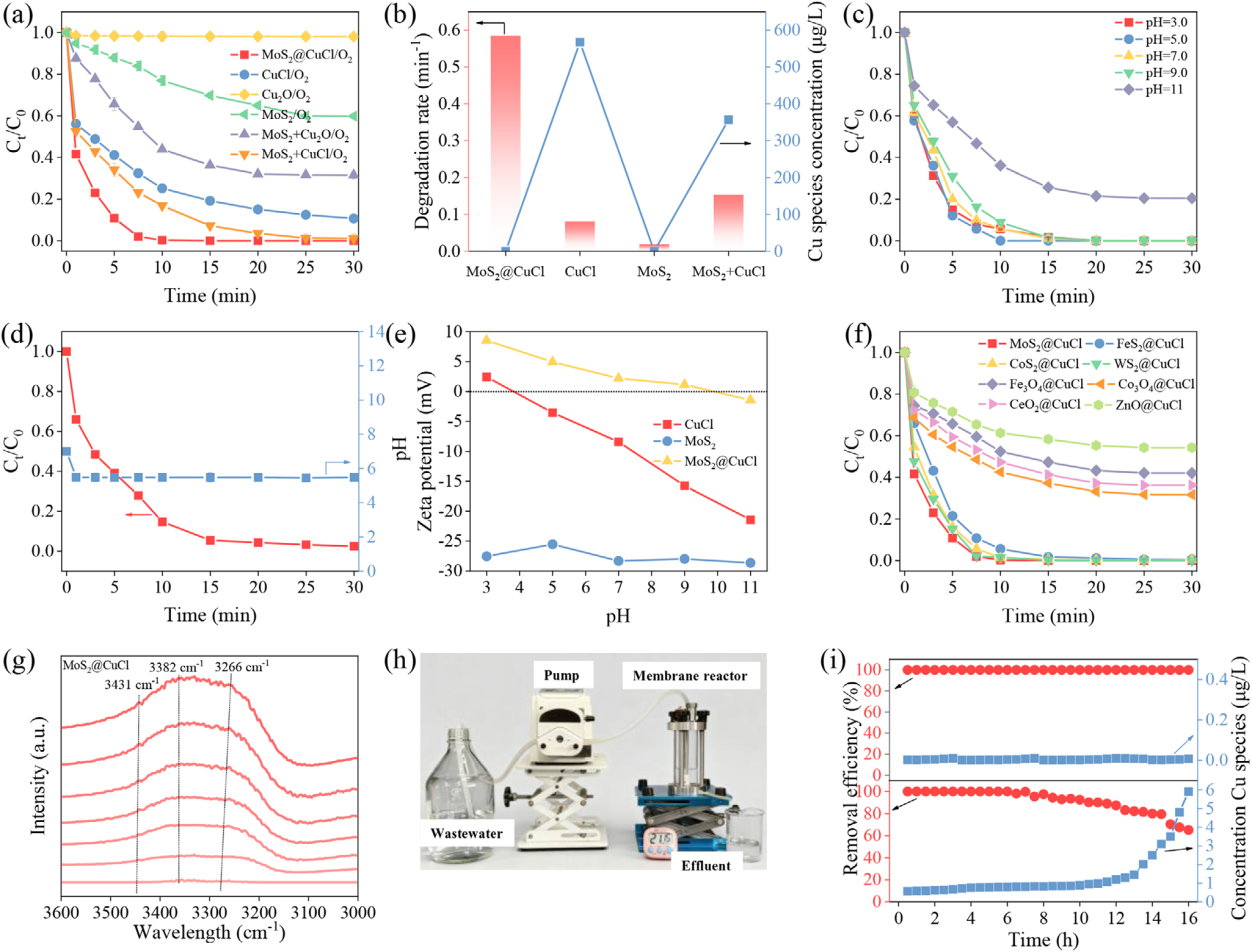
(a) TC degradation behavior of different catalysts at pH_initial_ ≈ 6.5, (b) degradation rate of TC and leached Cu species concentration, (c) degradation of TC at different initial pH values, (d) trend curve of TC degradation and the corresponding pH change in a neutral buffer solution, (e) Zeta potential of MoS_2_@CuCl under different pH conditions, (f) comparison of catalytic performances with the addition of metal sulfides or metal oxides, (g) in situ FTIR spectra of MoS_2_@CuCl in water, (h) the photo of TC degradation device and (i) pilot‐scale experiment.

The O_2_ activation capability of the MoS_2_@CuCl heterostructure was systematically evaluated using tetracycline (TC) as a model pollutant. Initial adsorption experiments showed that approximately 40% of TC was removed via hydrophobic interaction‐driven adsorption onto the catalyst surface (Figure S9). Notably, a volcano‐shaped relationship between MoS_2_ loading and catalytic activity indicated a non‐monotonic dependence, identifying the optimal composition as 1/20‐MoS_2_@CuCl (hereafter referred to as MoS_2_@CuCl). Under natural pH conditions (pH_initial_ ≈ 6.5), the optimized MoS_2_@CuCl catalyst achieved complete TC removal within 30 min (Figure 3a), representing a 3.9‐fold and 1.5‐fold enhancement over MoS_2_/O_2_ (65.6%) and CuCl/O_2_ (85.6%) systems, respectively. For a quantitative comparison of kinetics, the pseudo‐first‐order kinetic constant (k) of TC degradation was calculated (Figure 3b; Figure S10). Undoubtedly, the MoS_2_@CuCl/O_2_ system exhibited the best degradation rate constant, with a k value of 0.585 min^−1^, nearly 8.24 and 10.6 folds higher than those of MoS_2_ (0.071 min^−1^), and CuCl (0.055 min^−1^). This dramatic enhancement stems from the Cu‐S‐Mo interface, which couples electron transfer from MoS_2_ to Cu with a directional proton relay from S‐Hads sites, thereby accelerating the rate‐determining protonation of adsorbed *O_2_ to *OOH. Notably, this performance advantage remained significant even compared to previously reported catalytic systems that require additional oxidants (Table S2), highlighting the efficiency of this self‐driven O_2_ activation strategy.

The covalent Cu‐S‐Mo interface also imparts exceptional structural stability. Inductively coupled plasma‐mass spectrometry (ICP‐MS) analysis implied that the concentration of leached Cu species exhibited an inverse correlation with degradation efficiency. The MoS_2_@CuCl heterostructure suppressed Cu leaching to levels below the detection limit (< 0.1 µg/L), in stark contrast to pure CuCl (567.3 µg/L) and its physical mixture counterpart with MoS_2_ (356.8 µg/L) (Figure 3b). This pronounced improvement in copper retention demonstrated the crucial role of interfacial engineering in stabilizing CuCl under O_2_‐rich aqueous conditions (Figure S10). Importantly, while the physical mixture of MoS_2_ with CuCl also improved TC degradation performance, it failed to prevent substantial copper leaching (356.8 µg/L). Parallel experiments with Cu_2_O/O_2_ systems confirmed this trend, where the physically mixed MoS_2_+Cu_2_O combination showed similarly severe Cu leaching issues (Figure 3a; Figure S10). Thus, the covalently bonded interface was indispensable for both high activity and long‐term stability.

The MoS_2_@CuCl further exhibited outstanding pH adaptability, maintaining high TC degradation efficiency even at pH_initial_ ≈ 7.0 and pH_initial_ ≈ 9.0 (Figure 3c; Figure S11), whereas pure CuCl lost activity under alkaline conditions. This robustness was attributed to a localized acidic microenvironment created by proton storage on sulfur sites of MoS_2_. Direct evidence for this localized acidification was obtained through in situ pH monitoring. When the pH_initial_ of pollutant‐containing solutions was adjusted to neutral (≈7.0), the addition of MoS_2_@CuCl caused the system pH to approach ∼5.8. In addition, control experiments with MoS_2_ alone or with increased MoS_2_ content resulted in more pronounced pH reduction, directly verifying the proton‐regulating function of MoS_2_ and confirming the establishment of an acidic microenvironment at the catalyst interface (Figure 3d; Figure S12). To rigorously assess catalytic activity under truly neutral conditions while mitigating local pH effects, we conducted parallel experiments in a phosphate‐buffered medium that maintained a stable bulk pH of 7.0 throughout the reaction. Under these buffered conditions, MoS_2_@CuCl demonstrated significantly sustained catalytic activity, whereas the CuCl/O_2_ system achieved only 45.3% TC degradation within 30 min under identical conditions (Figure S13). This disparity confirmed that the exceptional catalytic efficiency of MoS_2_@CuCl arose from its ability to create a localized acidic microenvironment via S‐H_ads_ proton relay, overcoming the proton deficiency that plagued neutral O_2_ activation.

The interfacial acidic microenvironment was quantitatively probed by zeta potential measurements [26, 27]. While CuCl displayed characteristic pH‐dependent charge variations, MoS_2_@CuCl maintained remarkably stable zeta potential values throughout the pH range of 3.0 to 9.0 (Figure 3e), indicating a persistent proton supply mechanism. The Fourier transform infrared (FT‐IR) spectroscopy showed increased surface acidity for MoS_2_@CuCl (Figure S14). In situ diffuse reflectance infrared Fourier transform spectroscopy (DRIFTS) revealed that the O‐H stretching bands of hydrogen‐bonded water shifted to lower wavenumbers after introducing MoS_2_, indicating accelerated proton transfer dynamics, thereby enhancing the hydrogenation kinetics of *O_2_ to *OOH intermediates (Figure 3f) [28]. Overall, these results collectively confirmed that MoS_2_ generated a local acidic microenvironment enabling successive proton supply for O_2_ activation. The universality of the interfacial engineering was further validated across a series of metal sulfides. The corresponding composite catalysts prepared by coupling CuCl with CoS_2_, WS_2_, and FeS_2_ enabled efficient O_2_ activation for enhanced TC degradation and stability (Figure 3g), whereas Fe_3_O_4_, Co_3_O_4_, ZnO, and Al_2_O_3_ displayed very low activities, highlighting the unique proton regulation capability inherent to sulfur‐containing materials.

To fundamentally establish the oxygen‐dependent nature of the catalytic process, we systematically evaluated the TC degradation efficiency of MoS_2_@CuCl under different atmospheric conditions (air, O_2,_ and N_2_). As expected, the absence of O_2_ sharply decreased the TC removal rate of MoS_2_@CuCl, confirming that ROS was indeed generated from O_2_ activation (Figure S15). Crucially, the degradation performance under ambient air was nearly identical to that under pure O_2_ atmosphere, strongly underscoring the practical viability and energy autonomy of MoS_2_@CuCl for environmental applications driven directly by atmospheric O_2_. In addition to the catalytic performance, the environmental adaptability of the catalyst was also crucial for environmental remediation. The MoS_2_@CuCl exhibited extraordinary reactivity toward different contaminants, such as methylene blue (MB), rhodamine B (RhB), levofloxacin (LEV), Sulfamethoxazole (SMX), bisphenol A (BPA), and 4‐chlorophenol (4‐CP), which could be removed over 95% within 30 min, demonstrating its broad‐spectrum oxidative capability (Figure S16) [29]]. More importantly, this feature rendered the MoS_2_@CuCl/O_2_ system superior environmental robustness, as evidenced by the negligible interferences by various real water sources (tap water, river water, lake water and waste water) (Figure S16 and Table S3) and environmentally ubiquitous inorganic ions (e.g., F^−^, Cl^−^, H_2_PO_4_^−^, HCO_3_^−^, NO_3_^−^, CO_3_^2−^, and SO_4_^2−^) and metal ions (Na^+^, K^+^, Mg^2+^, Co^2+^, and Zn^2+^), implying the great practical application ability of the MoS_2_@CuCl (Figure S16).

To address large‐scale water treatment requirements, we engineered a flow‐through membrane reactor integrated with MoS_2_@CuCl for TC degradation (Figure 3h,i; Figure S17). The system maintained exceptional catalytic stability and treatment performance throughout extended continuous operation exceeding 16h, a remarkable improvement over the pure CuCl system, which suffered from rapid deactivation. Notably, the MoS_2_@CuCl/O_2_ system also achieved significant mineralization of TC, with a total organic carbon (TOC) removal rate of 56.8% within 30 min (Figure S17). The catalytic activity could be completely regenerated through simple ethanol washing to remove accumulated reaction intermediates, demonstrating both the structural integrity and operational recyclability of the MoS_2_@CuCl composite. The characterization after reaction confirmed that the material maintained its original morphological and structural features without detectable alteration, further confirming the robust nature of the covalently bonded Cu‐S‐Mo interface (Figures S18 and S19). This exceptional stability, coupled with the absence of membrane fouling, strongly supported the industrial scalability of the MoS_2_@CuCl/O_2_ system for sustainable water treatment [30, 31, 32].

**FIGURE 4:**
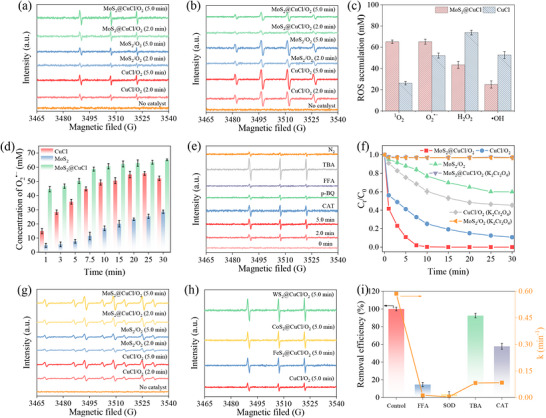
EPR signal of (a) TEMP‐^1^O_2_ and (b) DMPO‐•OH, (c) steady‐state concentration of O_2_^•−^, •OH, and H_2_O_2_, ^1^O_2_, (d) comparison and ratio of O_2_^•−^, •OH, H_2_O_2_, and ^1^O_2_, (e) EPR signal of TEMP‐^1^O_2_ intensity with other scavengers, (f) electron quencher experiments, (g) EPR signal of DMPO‐•H, (h) EPR signal of TEMP‐^1^O_2_ in different metal sulfides composites, and (i) the calculated ratio of individual ROS.

To elucidate the origin of the enhanced catalytic performance of MoS_2_@CuCl, we first used 2,2,6,6‐tetramethyl‐4‐piperidinyloxyl (TEMP) and 5,5‐dimethyl‐1‐pyrroline‐N‐oxide (DMPO) as spin traps to confirm ROS generation across all catalytic systems (Figure 4a; Figure S20) [33]. Notably, the intensities of the TEMP‐^1^O_2_ adduct signal for MoS_2_@CuCl were 2.4 times and 1.6 times stronger than those of the MoS_2_/O_2_ and CuCl/O_2_ systems, respectively. Besides, the characteristic peaks of the TEMP‐^1^O_2_ adduct in the MoS_2_@CuCl/O_2_ system gradually intensified with reaction time, indicating continuous production of ^1^O_2_ during operation. In contrast, the characteristic DMPO‐•OH signals in the CuCl/O_2_ system were significantly more intense than in the other systems (Figure 4b; Figure S20), indicating that the interfacial engineering modulated the selectivity of ROS generation. Further, we quantified ^1^O_2_ yield using 1,3‐diphenylisobenzofuran (DPBF) as a chemical probe (Figure 4c; Figure S21) [34, 35]. Strikingly, the ^1^O_2_ yield reached 65.19 mM for MoS_2_@CuCl within 30 min, substantially exceeding those of MoS_2_ and CuCl systems. Chemical probe analysis further quantified other ROS generation pathways with nitro‐blue tetrazolium chloride (NBT), benzoic acid (BA), and potassium iodide (KI) to verify the steady‐state concentrations of O_2_^•−^, •OH, and H_2_O_2_, respectively [36]. The free O_2_^•−^ generation efficiencies of pure CuCl, MoS_2_, and MoS_2_@CuCl were evaluated through O_2_ activation. As anticipated, MoS_2_@CuCl exhibited an ultrahigh production rate of O_2_^•−^ (2.17 mM/min), significantly surpassing that of pure MoS_2_ but was slightly higher than the performance of CuCl, which highlighted the critical role of low‐valent CuCl in facilitating O_2_ activation. Notably, in contrast to the slow O_2_^•−^ generation for CuCl, upon O_2_ introduction into the MoS_2_@CuCl system, a substantial steady‐state concentration of O_2_^•−^ was immediately detected (Figure 4d), signifying that the engineered interfacial acidic microenvironment dramatically accelerated the O_2_ activation kinetics. This rapid activation was further corroborated by accelerated dissolved oxygen (DO) depletion upon reaction initiation (Figure S21) [37]. Significantly, for the MoS_2_@CuCl/O_2_ system, •OH and H_2_O_2_ reached only 50.1% (24.93 mm) and 64.6% (43.38 mm) of those generated by the CuCl/O_2_ system, respectively (Figure 4d; Figure S21), further verifying the structural stability and rapid ^1^O_2_ generation ability of MoS_2_@CuCl [38, 39]. Collectively, these results demonstrated a fundamental redirection of O_2_ activation pathways; the Cu‐S‐Mo interface not only prevented O─O bond cleavage but also created an acid‐like microenvironment under neutral conditions, leading to enhanced ^1^O_2_ yield.

To check the ^1^O_2_ selectivity mechanism, we conducted a series of scavenging experiments targeting other possible ROS, including O_2_^•−^, •OH, and H_2_O_2_. The introduction of tert‐butanol (TBA, •OH scavenger) and catalase (CAT, H_2_O_2_ scavenger) caused a significant enhancement in the TEMP‐^1^O_2_ adduct signal intensity (Figure 4e), indicating a competitive relationship between ^1^O_2_ and •OH generation [40]. In contrast, the addition of p‐benzoquinone (p‐BQ, O_2_^•−^ scavenger) and furfuryl alcohol (FFA, ^1^O_2_ scavenger) completely suppressed the TEMP‐^1^O_2_ adduct signal, establishing O_2_^•−^ as the critical intermediate in the ^1^O_2_ generation pathway. This conclusion was further reinforced by time‐resolved EPR under N_2_‐saturated conditions (Figure S22), where the simultaneous almost disappearance of DMPO‐O_2_^•−^ and TEMP‐^1^O_2_ adduct signals confirmed their redox coupling. As shown in Figure S23, the DMPO‐O_2_^•−^ signal for MoS_2_@CuCl was 3.6‑fold stronger than for MoS_2_ and 1.4‑fold stronger than for CuCl, aligning with the highest ^1^O_2_ yield. To verify the involvement of •OOH, potassium dichromate (K_2_Cr_2_O_7_), an electron extractor that targets •OOH, was introduced [41, 42]. Remarkably, the addition of K_2_Cr_2_O_7_ substantially attenuated the TC degradation kinetics (Figure 4f), directly implicating •OOH as an essential precursor for ^1^O_2_ generation. Considering possible H_2_O_2_ generation via •OOH disproportionation, we added 3.0 mM H_2_O_2_ to the MoS_2_@CuCl system (higher than the maximum measured H_2_O_2_ concentration, Figures S21 and S24) but observed no ^1^O_2_ generation, excluding H_2_O2 as a source of ^1^O_2_.

Notably, the DMPO‐•OH signal was greatly enhanced with the addition of TC, but the TEMP‐^1^O_2_ adduct signal was obviously inhibited, further indicating the dominant role of ^1^O_2_ in the degradation process (Figure S25) [43]. As aforementioned, MoS_2_ could provide abundant proton relays to increase interfacial H^+^ concentration, while the interfacial protons are capable of trapping an electron to generate •H species (Cu^+^ + H^+^ → •H + Cu^2+^) [13, 14]. The detected EPR signal intensity was proportional to the *H_ads_ generation capacity of the catalysts [44]. As shown in Figure 4g and Figure S26, the MoS_2_@CuCl exhibited significantly stronger DMPO‐•H signal intensity compared to CuCl in the absence of O_2_. Conversely, the DMPO‐•H signal became nearly undetectable under O_2_‐rich conditions in the MoS_2_@CuCl system, suggesting that generated *H_ads_ were consumed for O_2_ hydrogenation to form the *OOH intermediate, which determined the ultimate ^1^O_2_ yield. This stark contrast underscored the critical role of the Cu‐S‐Mo bonds in steering electron transfer pathways toward direct ^1^O_2_ generation. Importantly, beyond the detailed investigation of MoS_2_@CuCl, we further examined whether other metal sulfides (WS_2_, CoS_2_, and FeS_2_) coupled with CuCl exhibited similar catalytic behavior for ^1^O_2_ generation. EPR spectra confirmed that all metal sulfides with Cu‐S‐Mo bonds promoted enhanced ^1^O_2_ generation rates and simultaneously stabilized the structure of CuCl (Figure 4h).

The scavenging experiments were further investigated to semi‐quantify the contributions of individual ROS. The low inhibition by TBA and CAT (<10%) in the MoS_2_@CuCl/O_2_ system (Figure 4i; Figure S27) indicated that •OH and H_2_O_2_ played only minor roles. In contrast, FFA dramatically inhibited TC degradation, confirming the crucial contribution of ^1^O_2_ (≥90%) [45]. Upon the addition of superoxide dismutase (SOD), the degradation was almost completely suppressed, similar to FFA, indicating that O_2_^•−^ is the critical intermediate for ^1^O_2_ generation [45]. Interestingly, replacing H_2_O with D_2_O suppressed TC degradation (Figure S28), contrary to the classical expectation that D_2_O should accelerate ^1^O_2_‐mediated reactions by extending the ^1^O_2_ lifetime [46]. This suppression reveals a normal kinetic isotope effect (k_H_/k_D_ > 1.0) on a proton‐coupled step, specifically the protonation of O_2_^•−^ to •OOH, which is rate‐limiting for ^1^O_2_ generation. This orthogonal kinetic evidence further supported the coupled electron‐proton transfer (CEPT) mechanism, demonstrating that the selective ^1^O_2_ production was governed by the interfacial proton relay rather than a simple energy‐transfer pathway. In contrast, for the CuCl/O_2_ system, both FFA and TBA caused substantial inhibition, with TBA being more effective (Figure S28), further highlighting that the effective ^1^O_2_ selectivity emerged specifically from the synergistic cooperation enabled by the Cu‐S‐Mo interfacial structure.

**FIGURE 5:**
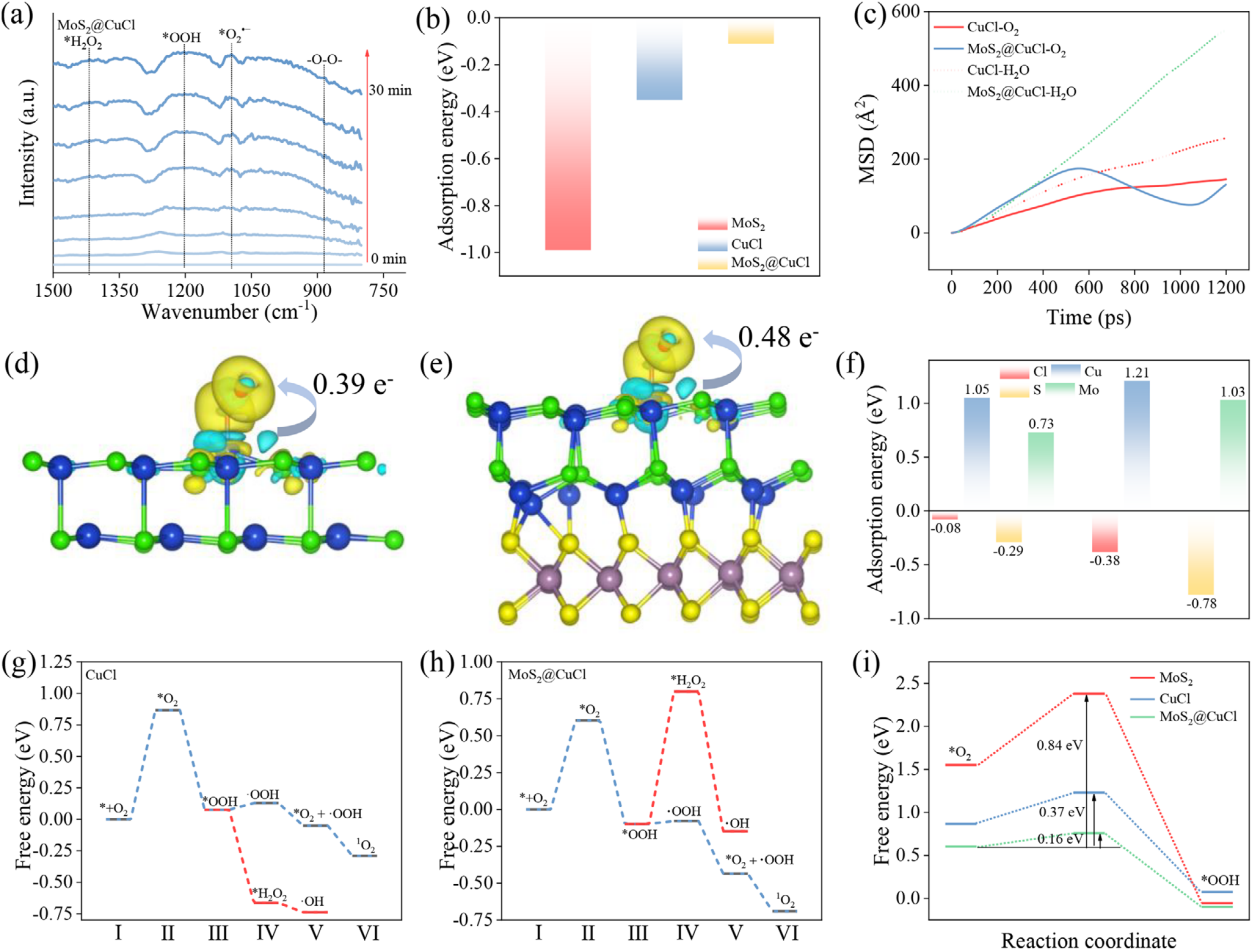
(a) In situ DRIFTS of CuCl/O_2_ and MoS_2_@CuCl/O_2_ systems, (b) calculated O_2_ adsorption energies of MoS_2_, CuCl and MoS_2_@CuCl, (c) MSD results, (d,e) O_2_ adsorption configurations and calculated differential charge densities, (f) adsorption energy of *H_ads_ on the different site of the MoS_2_, CuCl and MoS_2_@CuCl models, (g,h) calculated free‐energy diagrams of oxygen reduction pathways toward ^1^O_2_ production, and (i) the energy barrier of *O_2_ to *OOH on MoS_2_, CuCl and MoS_2_@CuCl.

To directly track the enhanced ^1^O_2_ selectivity during O_2_ activation, we conducted integrated characterizations including in situ Raman spectroscopy, DRIFTS, and density functional theory (DFT) calculations. This aligned with in situ Raman spectroscopy (Figure S29), where intensified peaks at around 900 cm^−1^ (*O_2_^•−^) on MoS_2_@CuCl confirmed the interfacial engineering mediated stabilization of critical intermediates [47]. The mediated electron‐transfer process was further evidenced by linear sweep voltammetry (LSV) and chronoamperometry (i‐t). The MoS_2_@CuCl electrode exhibited a higher current density in O_2_‐saturated electrolyte (Figure S29), directly evidencing enriched *OOH intermediates formation due to facilitated electron injection from the conductive carbon matrix [48]. Critically, in situ DRIFTS captured real‐time intermediates evolution under O_2_ flow (Figure 5a). Differential analysis showed increased absorption at approximately 1405, 1200, 1076, and 875 cm^−1^, corresponding to *H_2_O_2_, *OOH, *O_2_^•−^, and ─O─O─, respectively. These peaks exhibited transient volcano‐type intensity profiles that dynamically traced intermediate accumulation and consumption. To validate the assignment of *OOH, we performed an isotopic exchange experiment by replacing H_2_O with D_2_O during in situ DRIFTS (Figure S30). The significantly enhanced *H_2_O_2_ peak for CuCl further underscored that interfacial engineering modulated the ^1^O_2_‑selective intermediates (Figure 5b; Figure S30).

DFT calculations were performed to evaluate the O_2_ adsorption capabilities of different materials. As shown in Figure 5c and Figure S31, the adsorption energy for MoS_2_@CuCl was −0.11 eV, significantly higher (less negative) than those for MoS_2_ (−0.99 eV) and CuCl (−0.35 eV), indicating that the interfacial interaction weakened O_2_ chemisorption and thereby accelerated *OOH desorption. Previous studies have reported that strong interactions between unoccupied antibonding d‐orbitals of metal centers and 2p‐orbitals of O_2_^•−^/•OOH intermediates disfavored M─O bond cleavage, impeding desorption of O_2_^•−^/•OOH and their subsequent combination for ^1^O_2_ generation [49, 50]. Our results thus implied enhanced ^1^O_2_ selectivity over MoS_2_@CuCl, fully consistent with the scavenger experiments. Notably, the d‐band center position exhibited a nonlinear correlation with adsorption energy. As shown in Figure S32, CuCl possessed the most positive d‐band center value, which was favorable for the capture of O_2_ but simultaneously inhibited the *OOH desorption. The calculated charge density difference (CDD, Figure 5d,e; Figure S33) further revealed that electrons were depleted near Cu and accumulated near oxygen when O_2_ was absorbed. Compared with CuCl, MoS_2_@CuCl exhibited more charge depletion and accumulation at the catalyst‐O_2_ interface, demonstrating superior O_2_ activation capability through the construction of Cu‐S‐Mo channels. Moreover, the O─O bond length slightly elongated from initial 1.21 Å (initial O_2_) to 1.26 Å for MoS_2_@CuCl, which was significantly below the critical breaking threshold (>1.33 Å) for the 4e^−^ pathway and shorter than that on CuCl (1.29 Å), indicating that the MoS_2_@CuCl was more favorable for the O_2_ activation for *O_2_^•−^/*OOH generation (Figure S31) [51]. Notably, the interaction between MoS_2_ and O_2_ was weak, which was possibly attributed to the competitive adsorption process of O_2_ and *H_ads_ [15]. Overall, these results demonstrated that the interfacial microenvironment modulation of MoS_2_@CuCl significantly weakened O_2_ adsorption while preventing the O─O bond cleavage, both factored being conducive to selective ^1^O_2_ generation. Beyond electronic structure modulation, the MoS_2_@CuCl interface significantly enhanced reactant‑catalyst interactions [52]. The mean square displacement (MSD) curves implied much faster diffusion of TC and H_2_O on MoS_2_@CuCl, creating a high‑frequency collision environment. Notably, the O_2_ enrichment at the MoS_2_@CuCl interface was comparable to that on CuCl but much lower than on MoS_2_, suggesting that low‑valent Cu(I) was key for O_2_ activation, while rapid water diffusion provided a stable hydrogen‑bond network for proton transfer (Figure 5f; Figure S34) [28].

Gibbs free energy calculations were performed to gain deeper thermodynamic insights into reaction selectivity. These computations identified the free energy barrier for *O_2_ adsorption as the critical bifurcation point determining ^1^O_2_ generation pathways (Figure 5g,h; Figure S35). The ΔG value of *OOH→•OOH via a single‐electron pathway for MoS_2_@CuCl was calculated to be −0.08 eV, far lower than that of *OOH→*H_2_O_2_ (0.81 eV), revealing that the cleavage of ─M─O─ was thermodynamically favored. In contrast, for CuCl, the ΔG values of *OOH→*H_2_O_2_ (−0.66 eV) were far lower than that of *OOH→•OOH (0.12 eV), indicating the ^1^O_2_ production by low stable CuCl was also inefficient. The optimized *OOH structures further supported these thermodynamic findings; the M─O bond length in the case of *OOH on MoS_2_@CuCl was the longest among the samples, suggesting the relatively weaker adsorption of *OOH. Conversely, the O─O bond length in *OOH on MoS_2_@CuCl was calculated to be 1.31 Å, shorter than those on CuCl (1.45 Å) and MoS_2_ (1.48 Å). This shorter O─O bond effectively prevented the cleavage of the O─O bond in *OOH, ensuring high ^1^O_2_ selectivity (Figure S36). Importantly, for MoS_2_@CuCl, the reaction pathway from adsorbed *O_2_ to ^1^O_2_ generation was thermodynamically downhill in free energy, indicating a strong driving force for ^1^O_2_ production.

To further understand the ^1^O_2_ selectivity mechanism, the *H_ads_ mediated hydrogenation process of *O_2_ on MoS_2_@CuCl was explored in comparison with CuCl and MoS_2_. The adsorption energies of *H_ads_ on the possible sites (Mo^4+^, Cu^+^, Cl^−^, and S^2−^) were calculated. For MoS_2_@CuCl, the adsorption energy of *H_ads_ on S^2−^ was −0.29 eV, which was lower than those of Cl^−^ (−0.08 eV), Mo^4+^ (0.73 eV), and Cu^+^ (1.05 eV) (Figure 5h), suggesting the preferential *H_ads_ adsorption on S sites. Compared with MoS_2_@CuCl, the *H_ads_ adsorption for CuCl and MoS_2_ was significantly enhanced with a more negative adsorption energy of *H_ads_ on Cl^−^ sites (−0.38 eV) and S^2−^ (−0.78 eV), which indicated that the interfacial engineering between MoS_2_ and CuCl weakened the *H_ads_ adsorption by increasing the antibonding orbital occupancy of S‐H_ads_ bonds and promoting the desorption of *H_ads_. Figure 5i presented the transition state (TS) profiles of *O_2_ hydrogenation (*O_2_ + *H_ads_ → *OOH). The energy barrier from *O_2_ to TS was 0.16 eV for MoS_2_@CuCl, much lower than those for CuCl (0.37 eV) and MoS_2_ (0.84 eV), demonstrating that O_2_ hydrogenation is kinetically most favorable on MoS_2_@CuCl. Collectively, based on the results from electrochemical tests, in situ characterizations, and DFT calculations, we proposed that MoS_2_ played a crucial role in simultaneously modulating O_2_ adsorption and *H_ads_ supply to promote ^1^O_2_ production over MoS_2_@CuCl. Specifically, sulfur sites on MoS_2_ served as a proton sponge by forming S‐H_ads_ bonds, facilitating the directional migration of *H_ads_ via Cu‐S‐Mo channels to electron‐rich Cu sites, thereby accelerating the hydrogenation kinetics of *O_2_ to *OOH intermediates. Concurrently, the Cu sites enabled moderate binding strength for *O_2_ and *OOH, largely avoiding O─O bond cleavage and thereby enhancing ^1^O_2_ selectivity.

## 3 Conclusions

In summary, we have constructed a covalent Cu‐S‐Mo bridge between MoS_2_ and CuCl that creates a self‐adaptive acidic microenvironment, enabling spontaneous and efficient O_2_‐to‐^1^O_2_ conversion under neutral conditions without external energy. This local chemical environment circumvents the long‐standing proton‐transfer limitation in neutral ^1^O_2_ synthesis. Mechanistic studies reveal that electron‐deficient S sites on MoS_2_ act as a proton sponge via S‐H_ads_ bonds, while the covalent Cu‐S‐Mo channels facilitate directional proton relay to Cu sites. This architecture accelerates *O_2_ hydrogenation to *OOH, and concurrently, Cu sites moderate *O_2_/*OOH binding to promote *OOH desorption and suppress O─O cleavage, thereby steering selectivity toward ^1^O_2_. As a result, the system achieves rapid ^1^O_2_‐dominated degradation of refractory pollutants and sustained operation in a continuous‐flow membrane reactor. The design is broadly applicable to other metal sulfides, offering a versatile strategy for sustainable oxidant production. This work resolves the proton‐availability bottleneck in neutral ^1^O_2_ synthesis and establishes a design principle for microenvironment‐driven catalysis in water purification.

## 4 Methods

### 4.1 Materials

All the chemicals in this study were commercially available for the catalyst preparation and catalytic performance evaluation and are described in the Supporting Information.

## Author Contributions

**Qiaoyu Gao**: formal analysis, investigation, data curation, writing – original draft, methodology. **Xiaohui Dai**: methodology, software, data curation. **Chenxiao Yu**: validation, data curation. **Xiaohua Tian**: visualization, resources. **Yuehan Jiang**: methodology, formal analysis. **Lili Li**: validation, formal analysis. **Jun Zhao**: project administration, writing – review and editing, supervision, validation, resources. **Jiangdong Dai**: conceptualization, formal analysis, data curation, investigation. **Jianming Pan**: supervision, project administration, funding acquisition, resources. **Jian Ye**: writing – original draft, funding acquisition, validation, visualization, formal analysis.

## Conflicts of Interest

The authors declare no conflicts of interest.

## Supporting information




**Supporting File**: advs76027‐sup‐0001‐SuppMat.docx.

## Data Availability

The data that support the findings of this study are available from the corresponding author upon reasonable request.
