# Supplementary material for: Interfacial Proton‐Relay Microenvironment Enables Self‐Driven Singlet Oxygen Generation under Neutral Conditions
Source: Adv Sci (Weinh). 2026 Jun 10:e76027. Online ahead of print. doi: 10.1002/advs.76027 (PMC13337117; doi:10.1002/advs.76027)
Supplement: Supplementary file 1 — Supporting File: advs76027‐sup‐0001‐SuppMat.docx. [file ADVS-9999-e76027-s001.docx]

**Supporting Information**

**Interfacial Proton-Relay Microenvironment Enables Self-Driven Singlet Oxygen Generation under Neutral Conditions**

*Qiaoyu Gao, Xiaohui Dai, Jian Ye*, Lili Li, Chenxiao Yu, Yuehan Jiang, Jiangdong Dai*, Xiaohua Tian, Jun Zhao*, Jianming Pan**

Q. Gao, X. Dai, J. Ye, L. Li, C. Yu, J. Dai, X. Tian, Prof. J. Pan

School of Chemistry and Chemical Engineering, Jiangsu University, Zhenjiang 212013, China

E-mail: [yejian1211@ujs.edu.cn](mailto:yejian1211@ujs.edu.cn), daijd@ujs.edu.cn, pjm@ujs.edu.cn

J. Ye, Y. Jiang, Prof. J. Zhao

Department of Biology, Institute of Advanced Materials, Hong Kong Baptist University, Kowloon Tong, Hong Kong Special Administrative Region

E-mail: [zhaojun@hkbu.edu.hk](mailto:zhaojun@hkbu.edu.hk%20(J)

**1. Materials and methods**

**1.1. Materials**

The chemicals used for this experiment, copper mesh, sodium hydroxide (NaOH), hydrochloric acid (HCl), tert-butanol (TBA), methanol (MeOH), tetracycline (TC), atrazine (ATZ), bisphenol A (BPA), p-arsanilic acid (p-ASA), and Rhodamine B (RhB), p-nitrophenol (4-NP), ofloxacin (OFX), p-benzoquinone (p-BQ), ethanol, furfuryl alcohol (FFA), 5,5-dimethyl-1-pyrroline N-oxide (DMPO), 2,2,6,6-tetramethyl-4-piperidinol (TEMP), catalase (CAT), 1,3-diphenylisobenzofuran (DPBF), benzoic acid (BA), nitro blue tetrazolium (NBT), and coumarin were purchased from Aladdin Chemistry Co., Shanghai, China. Ultrapure water with a resistivity of 18.2 MΩ/cm from a M10illi-Q System was used for all experiments.

**1.2. Fabrication of CuCl and MoS_2_@CuCl**

**Synthesis of CuCl:** A certain amount of commercial Cu_2_O powder was dispersed in hydrochloric acid under stirring until complete dissolution was achieved. The resulting solution was then slowly dripped into deionized water. The precipitated solid particles were collected by centrifugation, followed by washing twice with N_2_-saturated deionized water and twice with ethanol. Finally, the product was dried under vacuum to obtain CuCl.

**​​Synthesis of MoS_2_@CuCl:** Pure MoS_2_ nanosheets were prepared by a hydrothermal method. In brief, 0.75 mmol MoCl_5_ and 3.0 mmol TAA were dissolved in 40 mL of deionized water to form a transparent solution with vigorous stirring for 2.0 h. Then, the mixture was poured into a 50 mL Teflon-lined autoclave and heated to 200 ℃ for 20 h. After dropping to room temperature, the product was washed several times with DI water and absolute ethanol, followed by drying at 50 ℃ overnight. A certain amount of MoS₂ was dispersed in N₂-saturated deionized water under continuous stirring. Subsequently, a pre-prepared Cu_2_O-HCl solution was added dropwise into the above dispersion to facilitate a self-assembly process. This procedure ultimately yielded the MoS_2_@CuCl composite. For comparison, MoS_2_@CuCl with different mass ratios can be synthesized by controlling the mass of MoS_2_.

**1.3. Experimental Procedure**

TC degradation trials employed both a membrane reactor and a traditional suspension system. In the suspension method, the catalyst was first mixed with the TC solution for 30 min to ensure adsorption-desorption equilibrium. The degradation reaction was then initiated by a continuous flow of O_2_. During this process, 1.0 mL samples were periodically extracted and immediately filtered through a 0.22 μm nitrocellulose membrane with methanol to halt the reaction, allowing for subsequent analysis.

Removal efficiency (R, %) of pollutants by the MoS_2_@CuCl/O_2_ systems can be calculated as follows:

$R=\frac{C_{f}-C_{p}}{C_{f}}$ (S2)

where C_P_ and C_f_ are the concentrations of the organic pollutants in permeate and feed, respectively. Statistical Analysis: Statistical analysis of data presented in this work had a sample size n = 3.0 and was presented as the mean ± SD. The statistical analyses were carried out with the OriginPro 2018 software.

**2. Characterization**

The structures of samples were determined by X-ray diffractometer (XRD, Rigaku, UltimaIV) with Cu Kα source irradiation. The surface morphologies and inner structures of the samples were analyzed by scanning electron microscopy (SEM, Zeiss, Sigma 500) and transmission electron microscopy (TEM, JEOL JEM-2010). The. Fourier transform infrared (FT-IR) spectroscopy was performed in the spectral range of 4000-500 cm^-1^. An X-ray photoelectron spectroscopy (XPS) system (PHI 5000 Versa probe spectrometer) was used to study the chemical states of the catalyst and calibrated using the C 1s peak at 284.8 eV. Electron paramagnetic resonance (EPR) tests were performed on a Bruker EMX-E8/2.7 spectrometer with DMPO and TEMP as the spin-trapping agent. Fe and Co leaching was measured by inductively coupled plasma-optical emission spectroscopy (ICP-OES) on Agilent 720ES. The X-ray absorption near-edge structure (XANES) and extended X-ray absorption fine structure (EXAFS) of Co and Fe K-edge were measured at the BL14W1 beamline of the Shanghai Synchrotron Radiation Facility (SSRF). The XANES and EXAFS data were processed and analyzed with ATHENA and ARTEMIS software.

**3. Identification of ROS**

In the radical quenching experiments, tertiary-butyl alcohol (TBA) for •OH, potassium iodide (KI) for surface-bound radicals, and furfuryl alcohol (FFA) and sodium azide (NaN_3_) for ^1^O_2_.[1] In addition, we replaced the water solution with D_2_O to further validate the ^1^O_2_ production.^[2]^ The contributions of •OH and ^1^O_2_ for the TC degradation were quantified based on the discrepancy of the degradation kinetic constant in the presence of different scavengers.

**4. ROS concentration measurements**

**·OH concentration measurement.** Benzoic acid (BA) was used as a molecular probe to detect •OH concentration. The aeration tube was immersed in 100.0mL of BA aqueous solution (10.0mM) before the reaction, and oxygen content in the solution was controlled by aeration with air and pure oxygen. The BA solution was added to the reactor to initiate the reaction. At predetermined time intervals, 1.0 mL of the suspension was taken and filtered through a 0.22 μm nitrocellulose membrane with methanol as the quencher of the reaction. The reaction of BA with •OH can produce p-HBA, and •OH concentration was analyzed by p-HBA concentration according to the equation: [•OH] = [p-HBA] × 5.87. The p-HBA concentration was measured by a UV-vis spectrophotometer at 270 nm. Three parallel experiments were carried out for each group of samples.

**O_2_^•−^ concentration measurement.** Nitrotetrazolium blue chloride (NBT) was used as the molecular probe of O_2_^•−^. The aeration tube was immersed in 100.0mL of NBT aqueous solution (1.0mM) before the reaction, and oxygen content in the solution was controlled by aeration with air and pure oxygen. The NBT solution was added to the reactor to initiate the reaction. At predetermined time intervals, 1mL of the suspension was taken and filtered through a 0.22μm nitrocellulose membrane. The yellow NBT was reduced to blue formazan by O_2_^•−^, and the concentration formula was [O_2_^•−^] = [formazan] × 4. The concentration change of NBT was measured at 259 nm with a UV-vis spectrophotometer. Three parallel experiments were carried out for each group of samples.

**^1^O_2_ concentration measurement.** Using 1,3-diphenylisobenzofuran (DPBF) as an indicator of ^1^O_2_. The aeration tube was immersed in 100.0 mL of DPBF ethanol solution (0.2mM) before the reaction, and oxygen content in the solution was controlled by aeration with air and pure oxygen. The DPBF solution was added to the reactor to initiate the reaction. At predetermined time intervals, 1.0 mL of the suspension was taken and filtered through a 0.22μm nitrocellulose membrane. The chemical reaction molar ratio of ^1^O_2_ to DPBF was 1:1, and the concentration change of DPBF was measured at the wavelength of 410 nm. Three parallel experiments were carried out for each group of samples.

**H_2_O_2_ concentration measurement.** The produced H_2_O_2_ was quantified by reagent color-developing method. First, 1.0 mL filtered reaction solution was attenuated by 2.0 mL DI water, after that, 1.0 mL of the attenuated sample was added into a mixed solution of 1.0 mL KI (0.4 M) aqueous solution and 1.0 mL commercial pH buffer with pH=4.0 (potassium biphthalate). Finally, the solution was kept for 1.0h in dark. The H_2_O_2_ concentration can be determined by the triiodide anions (I_3_^-^) concentration according to the following equation, where the I3- can be estimated by Lambert-Beer's law due to their strong absorbance at 350 nm.

**Figure S1.** **Characterization analysis of TEM.** (a) and (b) HRTEM images of MoS_2_@CuCl, and (c) SAED pattern of MoS_2_@CuCl.

**NOTE:** The SAED pattern shows three discernible diffraction rings indexed to CuCl (111), (200), and (220). A very weak ring at ~0.62 nm, which would correspond to MoS_2_ (002), is barely visible due to the low MoS_2_ loading (~5.0 wt%), its few‑layer thickness, and its flat‑lying orientation on the TEM grid. The presence of MoS_2_ is unequivocally confirmed by HRTEM (0.617 nm lattice fringes), Raman, XPS, and EXAFS. Thus, SAED serves primarily to confirm the CuCl phase; the weakness of the MoS_2_ ring does not contradict its presence but rather reflects the designed structural features.

**Figure S2.** **Characterization analysis of TEM and SEM.** HRTEM images of (a) and (b) MoS_2_, and (c) and (d) CuCl, and SEM images of (c) MoS_2_ and (f) CuCl.


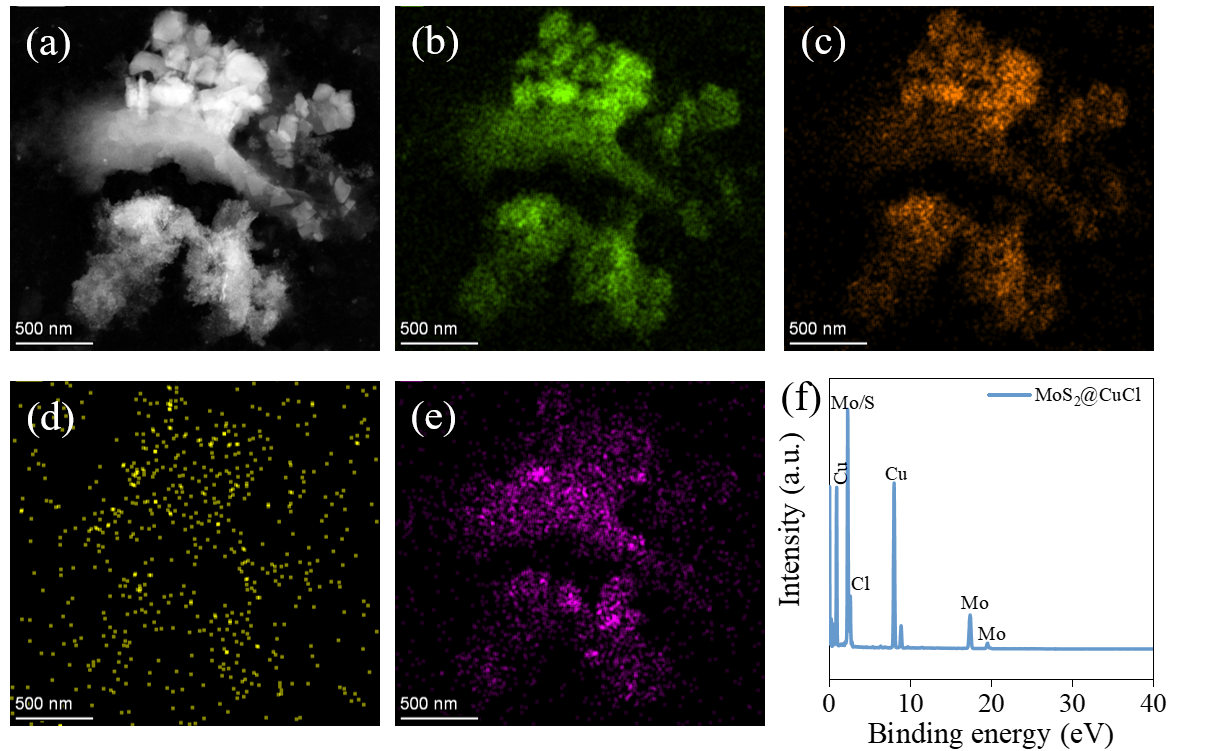


**Figure S3.** **Characterization analysis of TEM.** Element mapping of (a)-(e) MoS_2_@CuCl, and (f) element contents.

**Figure S4. Characterization analysis of XRD and Raman spectra.** (a) XRD patterns of MoS_2_@CuCl with different mass ratios, and (b) Raman spectra of MoS_2_ and MoS_2_@CuCl.

**Figure S5. XPS spectra.** The XPS of (a) the full survey scan, (b) Cl 2p, (c) Mo 3d, and (d) Cu LEM.

**Figure S6. Electrochemical analysis.** EIS spectra of MoS_2_, CuCl, and MoS_2_@CuCl.

**Figure S7. EXAFS fitting curve.** The fitting curves of k3-weighted EXAFS spectra of (a) Cu_2_S, (b) Cu foil, (c) Cu_2_O, (d) CuCl, (e) CuO, and (f) MoS_2_@CuCl.

**Figure S8. EXAFS characterization.** (a)-(f) Cu K-edge EXAFS fitting curves of Cu_2_S, Cu foil, Cu_2_O, CuCl, CuO, and MoS_2_@CuCl at K-space.

**Figure S9. Degradation performance.** Effect of (a) TC degradation with different MoS_2_ loading, (b) catalyst loading in MoS_2_@CuCl/O_2_ systems, (c) degradation performance of MoS_2_@CuCl under O_2_ and N_2_ conditions, (d) degradation and adsorption performance of MoS_2_@CuCl, (e) and (f) contact angle of MoS_2_ and MoS_2_@CuCl, (g), (h) and (i) UV-Vis spectra of MoS_2_@CuCl under N_2_, O_2_ or N_2_ + O_2_ conditions.

**NOTE:** To deconvolute the contribution of adsorption from oxidative degradation, a control experiment was performed under an N_2_ atmosphere. As shown in Figure S9c, the MoS_2_@CuCl composite adsorbed approximately 40% of the initial TC within 30 min, reaching equilibrium. Under an O_2_ atmosphere, the removal profile encompasses this initial adsorption phase followed by continuous catalytic oxidation, with the latter being the dominant removal pathway (>60%). The adsorption of TC onto the catalyst surface likely enhanced the interfacial reaction efficiency by concentrating the pollutant near the active sites.

**Figure S10. Degradation performance.** Effect of (a) different oxidants and (b) MoS_2_ in CuCl/O_2_ or Cu_2_O/O_2_ systems, (c) and (d) comparison of degradation rate.

**Figure S11. Effect of pH.** (a) and (b) effect of pH in the CuCl/O_2_ and MoS_2_/O_2_ systems.

**NOTE:** To evaluate the structural stability under different pH conditions, Cu leaching from MoS2@CuCl and pure CuCl was quantified (Figure S11c). The MoS₂@CuCl composite demonstrated remarkable stability, with Cu leaching maintained below 8 µg/L across pH 3.0-11. In contrast, pure CuCl exhibited severe pH-dependent leaching, exceeding 1.0 mg/L under acidic conditions (pH 3.0). This result confirms that the covalent Cu-S-Mo interface effectively immobilizes copper species, which is the foundation for the composite's broad pH adaptability and sustained catalytic activity

**Figure S12. Solution pH. (**a) and (b) variation of solution pH in the CuCl/O_2_ and MoS_2_/O_2_ systems.

**Figure S13. Solution pH and degradation performance.** (a) and (b) the correalation between degradation performance and solution pH, and (c) degradation peformance with the presence of buffer solution.

**Figure S14. FTIR spectra.** (a) FTIR spectra of MoS_2_, CuCl and MoS_2_@CuCl, and (b) in-situ FTIR spectra of CuCl with the absence of O_2_.

**Figure S15. Degradation performance.** Degradation performance of the MoS_2_@CuCl/O_2_ system under different conditions with O_2_, N_2_ or air.

**Figure S16. Degradation performance.** Effect of (a) various pollutants, (b) inorganic ions, (c) metal ions, and (d) various water bodies in the MoS_2_@CuCl/O_2_ system.

**Figure S17. Degradation performance and TOC removal.** (a) and (b) degradation of TC and TOC removal in the MoS_2_@CuCl/O_2_ and CuCl/O_2_ systems, and (c) the degradation pathway of TC in the MoS_2_@CuCl/O_2_ system.

​**NOTE:** The pathway clearly shows that ^1^O_2_ from Figure S17c, generated by our catalyst, selectively attacks the electron-rich aromatic rings and C=C bonds of TC via electrophilic addition. This leads to stepwise hydroxylation, demethylation, and ring-opening reactions, breaking down the complex molecule into smaller, oxygenated aliphatic fragments. The terminal products of this ^1^O_2_-driven pathway are identified as short-chain carboxylic acids (C1-C4), such as formic, acetic, and oxalic acids. This is a well-documented endpoint for ^1^O_2_ oxidation of complex organics.

**Figure S18. Characterization and performance.** (a) XRD patterns of MoS_2_, CuCl, and MoS_2_@CuCl before and after reaction, and (b) SEM image of MoS_2_@CuCl after reaction, (c) variation of pH during long-term stability, (d) Zeta potential of used MoS_2_@CuCl and CuCl under different pH conditions, (e) XANES spectra at the Cu K-edge, (f) FT k3-weighted EXAFS spectra of MoS_2_@CuCl, used MoS_2_@CuCl and CuCl.

**NOTE:** As shown in the Figure S18c and S18d, the used catalyst maintains a similarly negative zeta potential profile​ across pH 3.0-11 as the fresh catalyst. Its point of zero charge (PZC) was unchanged and remained significantly lower than that of pure CuCl, which confirmed that the surface acidic property, engineered by the MoS_2_ proton reservoir, was preserved.

**Figure S19. XPS spectra.** The XPS of (a) the full survey scan, (b) Mo 3d (c) Cu 2p, (d) Cu LEM, (e) S 2s, and (f) Cl 2p.

**Figure S20. EPR spectra.** The signal of (a) TEMP-^1^O_2_ and (b) DMPO-•OH.

**Figure S21. ROS concentration and DO consumption.** ROS concentration of (a) ^1^O_2_, (b) H_2_O_2_ and (c) •OH, and DO consumption in the MoS_2_/O_2_, CuCl/O_2_ and MoS_2_@CuCl/O_2_ systems.

**Figure S22. EPR spectra.** The signal of (a) TEMP-^1^O_2_ and (b) DMPO-O_2_^•−^ in the MoS_2_@CuCl/N_2_ systems.

**Figure S23. EPR spectra.** The signal of (a)-(d) DMPO-O_2_^•−^ in the MoS_2_@CuCl/O_2_, MoS_2_/O_2_, and CuCl/O_2_ systems.

**Figure S24. Degradation performance.** Degradation performance and ^1^O_2_ accumulation MoS_2_@CuCl/H_2_O_2_ systems.

**NOTE:** According to the literature, if the activation of O_2_ for ^1^O_2_ production by the MoS_2_@CuCl only existed in a two-step one-electron process (O_2_→ O_2_^•−^/•OOH → ^1^O_2_), the O_2_^•−^ generated by O_2_ activation would not produce H_2_O_2_ and •OH after being captured.^[3]^ The amount of H_2_O_2_, •OH, O_2_^•−,^ and ^1^O_2_ produced in the MoS_2_@CuCl with p-BQ was negligible, which implied that the simple-electron transfer of O_2_ dominated the production of ROS. However, the trace H_2_O_2_ was detected in the CuCl/O_2_ system, which could be explained that the dissolved Cu^+^ reacted with O_2_ via the direct two-electron process.[2b] In addition, we first monitored the TC degradation and ROS generation with the addition of H_2_O_2_ in the MoS_2_@CuCl system, and the main source of ^1^O_2_ came from the disproportionation reaction of O_2_^•−^/•OOH, which further verified the ^1^O_2_-dominated pathways. Further,

**Figure S25. EPR spectra.** The signal of (a) TEMP-^1^O_2_ with or without TC, and (b) DMPO-O_2_^•−^ with or without TC in the MoS_2_@CuCl/O_2_ systems.

**Figure S26. EPR spectra.** The signal of DMPO-•H in the MoS_2_@CuCl/O_2_ systems.

**Figure S27. Quencher experiments.** Effect of (a) TBA, (b) CAT, (c) SOD, and (d) FFA in the MoS_2_@CuCl/O_2_ systems.

**Figure S28. Role of ^1^O_2_ and quencher experiments.** (a) Degradation performance of TC with H_2_O or D_2_O as solvents in the MoS_2_@CuCl/O_2_ systems, and (b) quencher experiments in the CuCl/O_2_ systems.

**NOTE:** Degradation is suppressed in D_2_O, opposite to the classical acceleration effect expected for ^1^O_2_‑mediated reactions.^[4]^ This suppression arises from a normal kinetic isotope effect on the rate‑determining protonation of O_2_^•⁻^ to OOH (O_2_^•⁻^ + H^+^ → *OOH). The slower deuteration in D_2_O reduces *OOD formation and lowers ^1^O_2_ production, proving that the reaction follows a coupled electron‑proton transfer (CEPT) mechanism with a proton‑coupled rate‑determining step.

**Figure S29. Electrochemical analysis.** (a) In-situ Raman spectra of MoS_2_@CuCl, MoS_2_@CuCl/O_2_ and CuCl/O_2_ systems, (b) LSV spectra, and (c) EIS spectra in the MoS_2_@CuCl/O_2_ systems.

**Figure S30. In-situ FTIR spectra.** In-situ FTIR spectra of (a) MoS_2_@CuCl/O_2_ with D_2_O as solvent, and (b) CuCl/O_2_ with the prolonged time.

**NOTE:** In the D_2_O experiment (Figure S30a), the O-D bending band (~1180 cm^-1^) is nearly undetectable during the first few minutes, whereas the O‑O stretching band (~875 cm^-1^) appears weakly after a short induction period. This is attributed to the normal kinetic isotope effect (k_H_/k_D_ > 1) on the rate‑determining proton transfer step (*O_2_^•⁻^ + H^+^ → *OOH). The slower formation of *OOD in D_2_O lowers its surface coverage below the IR detection threshold initially. As the reaction proceeds (>5.0 min), *OOD gradually accumulates, and its bending band becomes discernible. This observation, together with the slower TC degradation rate in D_2_O (Figure S28a), provides direct kinetic evidence that the proton‑coupled step is rate‑limiting. In contrast, in H_2_O (Figure 5a), the *OOH bending band appears immediately, consistent with faster formation kinetics.

**Figure S31. DFT calculation.** Adsorption energy of (a) MoS_2_, (b) CuCl, and (c) MoS_2_@CuCl.

**Figure S32. PDOS spectra.** PDOS of (a) MoS_2_@CuCl/O_2_, (b) CuCl/O_2_, and (c) MoS_2_/O_2_ systems.

**Figure S33. Differential charge density.** (a)-(c) O_2_ adsorption configurations and calculated differential charge densities.

**Figure 34. MSD simulation.** MSD results showing reactant diffusion kinetics for different catalysts

**Figure S35. Adsorption energy.** The energy barrier of *O_2_ to ^1^O_2_ on MoS_2_.

**Figure S36.** Adsorption Bond Length. The adsorption bond length of *OOH on MoS_2_, CuCl, and MoS_2_@CuCl.

**Table S1.** EXAFS fitting parameters for various samples.

| **Sample** | **Shell** | ***CN^a^*** | ***R*(Å)*^b^*** | **σ^2^(Å^2^·10^-3^*)^c^*** | | **Δ*E*_0_(eV)*^d^*** | **R factor (%)** |
| --- | --- | --- | --- | --- | --- | --- | --- |
| Cu-foil | Cu-Cu | 12* | 2.54±0.01 | 0.0086 | 4.1±0.05 | | 0.004 |
| CuO | Cu-O | 4* | 1.94±0.01 | 0.0053 | 2.0±0.9 | | 0.012 |
|  | Cu-Cu | 4* | 2.96±0.03 | 0.0031 |  |  |  |
| Cu_2_O | Cu-O | 2* | 1.85±0.02 | 0.0018 | 7.2±0.9 | | 0.019 |
|  | Cu-Cu | 12* | 3.02±0.02 | 0.0025 |  |  |  |
| MoS_2_@CuCl  (Before) | Cu-Cl | 3.26±0.6 | 2.23±0.09 | 0.0027 | 1.5±1.1 | | 0.007 |
|  | Cu-S | 3.03±0.5 | 2.40±0.09 | 0.0024 |  |  |  |
| MoS_2_@CuCl (After) | Cu-Cl | 3.31±0.6 | 2.21±0.04 | 0.0021 | 1.4±0.8 | | 0.008 |
|  | Cu-S | 3.01±0.7 | 2.46±0.06 | 0.0023 |  |  |  |
| CuCl | Cu-Cl | 4* | 2.29±0.03 | 0.0053 | 2.2±1.0 | | 0.011 |
| Cu_2_S | Cu-S | 4* | 2.39±0.01 | 0.0090 | 4.2±0.8 | | 0.003 |
|  | Cu-Cu | 6* | 2.67±0.09 | 0.0096 |  |  |  |

*^a^* *N*: coordination numbers; *^b^* *R*: bond distance; *^c^* *σ*^2^: Debye-Waller factors to account for both thermal and structural disorders; *^d^* Δ*E*_0_: the inner potential correction. *R* factor: goodness of fit.

*Ѕ*_0_^2^ was set as 0.94 for Cu data. This was obtained from the experimental EXAFS fit of the mental foil reference by fixing CN as the known crystallographic value, and was fixed to all the samples.

**Table S2.** H_2_O_2_, PMS, and O_2_ reactions for TC degradation.

| No. | Methods | Materials | Catalyst dosage  (mg/L) | Pollutant concentration  (mg/L) | Pollutant removal efficiency | Reaction time | k (min^-1^) | Ref |
| --- | --- | --- | --- | --- | --- | --- | --- | --- |
| 1 | H_2_O_2_ activation | Fe-BC | 200 | 20 | 100% | 60 | 0.163 | [5] |
| 2 | H_2_O_2_ activation | α-Fe_2_O_3_ | 600 | 10 | < 95% | 120 | 0.061 | [6] |
| 3 | H_2_O_2_ activation | CuS@PDA | 100 | ~20 | < 95% | 50 | 0.051 | [7] |
| 4 | H_2_O_2_ activation | CoFe2O4 | 300 | 50 | < 90% | 50 | 0.041 | [8] |
| 5 | PMS activation | CoP_x_@NC | 100 | 10 | < 95% | 30 | 0.137 | [9] |
| 6 | PMS activation | N-CoS_2_@C | 60 | 20 | < 95% | 11 | 0.194 | [10] |
| 7 | PMS activation | Cu_2_O/Cu@MXene | 200 | 20 | 100% | 30 | 0.151 | [11] |
| 8 | PMS activation | Co_3_O_4_/CPANI | 150 | 20 | < 95% | 40 | 0.090 | [12] |
| 9 | PMS activation | NC-Fe_2_O_3_(II) | 200 | 20 | < 95% | 10 | 0.176 | [13] |
| 10 | PMS activation | α-FeOOH | 100 | 10 | < 95% | 10 | 0.188 | [14] |
| 11 | PMS activation | CuCo@GCN-3 | 100 | 20 | < 95% | 30 | 0.182 | [15] |
| 12 | PMS activation | Co-N/KC-900 | 160 | 20 | 100% | 24 | 0.196 | [16] |
| 13 | PMS activation | Co@NCNTs-600 | 120 | 20 | < 95% | 20 | 0.123 | [17] |
| 14 | PMS activation | FeCoNC-2 | 300 | 80 | 100% | 30 | 0.011 | [18] |
| 15 | PMS activation | [MC@NCF](mailto:Co3O4@Co@rGO) | 75 | 20 | 100% | 10 | 0.523 | [19] |
| 16 | O_2_ activation | MoS_2_@CuCl | 25 | 10 | 100% | 30 | 0.587 | This work |

**Table S3.** The basic water quality parameters of different water matrices.

| Water quality parameters | DI water | Yudai River water | Seawater  From Hong Kong | Rainwater | Tap water | Wastewater Treatment Plant |
| --- | --- | --- | --- | --- | --- | --- |
| pH |  | 6.85 | 5.43 | 5.28 | 6.12 | 5.11 |
| Suspended solids (mg/L) | — | 20.00 | 6.73 | 4.09 | 17.76 | 32.65 |
| Turbidity | 0.22 | Not detected | Not detected | Not detected | Not detected | Not detected |
| Chroma | 5.00 | 2.10 | 4.32 | 3.65 | 2.98 | 2.16 |
| Ammonia nitrogen (mg/L) | — | 1.38 | 5.49 | 1.38 | 3.76 | 5.58 |
| Total nitrogen (mg/L) | — | 3.18 | 5.67 | 4.75 | 4.23 | 6.77 |
| Total phosphorus (mg/L) | — | 0.63 | 2.65 | 2.11 | 1.59 | 4.56 |
| COD (mg/L) | 1.10 | 45.00 | 69.87 | 58.09 | 49.88 | 87.60 |
| Free chlorine residual | 0.50 | — | 3.34 | 2.41 | 1.76 | 2.65 |

**^a^** The data provided by Zhenjiang Water Works Company.

**^b^** The data provided by Shiyanjia Lab.

**References**

[1] a) Z.-Y. Guo, R. Sun, Z. Huang, X. Han, H. Wang, C. Chen, Y.-Q. Liu, X. Zheng, W. Zhang, X. Hong, W.-W. Li, *P. Nat. A. Sci.* **2023**, *120* (15), e2220608120; b) X. Liang, D. Wang, Z. Zhao, T. Li, Y. Gao, C. Hu, *Adv. Funct. Mater.* **2022**, *32* (38), 2203001.

[2] a) X. Liu, Y. Liu, H. Qin, Z. Ye, X. Wei, W. Miao, D. Yang, S. Mao, *Environ. Sci. Technol.* **2022**, *56* (4), 2665; b) S. Wang, J. Zhu, T. Li, F. Ge, Z. Zhang, R. Zhu, H. Xie, Y. Xu, *Environ. Sci. Technol.* **2022**, *56* (12), 7924; c) Z. Zhao, P. Wang, C. Song, T. Zhang, S. Zhan, Y. Li, *Angew. Chem. Inter. Edit.* **2023**, *62* (11), e202216403.

[3] Z. Yang, Z. Wang, J. Wang, Y. Li, G. Zhang, *Environ. Sci. Technol.* **2022**, *56* (24), 18008.

[4] S. Garg, T. D. Waite, *Environ. Sci. Technol.* **2025**, *59* (49), 26320.

[5] X. Li, Y. Jia, J. Zhang, Y. Qin, Y. Wu, M. Zhou, J. Sun, *Chinese Chem. Lett.* **2022**, *33* (4), 2105.

[6] M. Shi, W. Tang, S. Guo, F. Chen, *Environ. Res.* **2025**, *286*, 122854.

[7] Z. Shi, C. He, H. Huang, X. Huang, T. Hu, Y. He, D. Yang, S. Xia, H. Zhang, L. Deng, *J. Water Process Eng.* **2025**, *71*, 107223.

[8] M. Nie, Y. Li, L. Li, J. He, P. Hong, K. Zhang, X. Cai, L. Kong, J. Liu, *Appl. Surf. Sci.* **2021**, *535*, 147655.

[9] L. Ai, S. Yang, W. Wei, Y. Li, X. Zheng, J. Jiang, *Sep. Purif. Technol.* **2023**, *322*, 124257.

[10] S. Liu, J. Cheng, A. Guo, G. Fan, *Environ. Pollut.* **2023**, *330*, 121761.

[11] Q. Zhou, P. Hong, X. Shi, Y. Li, K. Yao, W. Zhang, C. Wang, J. He, K. Zhang, L. Kong, *J. Hazard. Mater.* **2023**, *448*, 130995.

[12] J. Qian, X. Mi, Z. Chen, W. Xu, W. Liu, R. Ma, Y. Zhang, Y. Du, B.-J. Ni, *J. Cleaner Prod.* **2023**, *405*, 137023.

[13] M. Wang, S. Li, J. Kang, Y. Tang, J. Wang, Z. Xu, J. Liu, *Chem. Eng. J.* **2023**, *451*, 138611.

[14] Y. Han, Z. Li, M. Zhang, F. Han, Z. Liu, W. Zhou, *Chem. Eng. J.* **2022**, *450*, 138460.

[15] L. Liu, C. Han, G. Ding, M. Yu, Y. Li, S. Liu, Y. Xie, J. Liu, *Chem. Eng. J.* **2022**, *450*, 138302.

[16] H. Zhu, A. Guo, S. Wang, Y. Long, G. Fan, X. Yu, *Chem. Eng. J.* **2022**, *450*, 138428.

[17] L. Hu, Y. Zhang, X. Liu, H. Zhu, J. Wu, Y. Wang, Y. Long, G. Fan, *Chem. Eng. J.* **2022**, *450*, 138219.

[18] B. Zhang, X. Li, P. A. Bingham, K. Akiyama, S. Kubuki, *Chem. Eng. J.* **2023**, *451*, 138574.

[19] J. Ye, J. Yang, Y. Liu, W. Xue, J. W. C. Wong, J. Dai, J. Zhao, J. Crittenden, *Chem. Eng. J.* **2024**, *496*, 154034.
